# Pilot Malacology Surveys for the Intermediate Hosts of Schistosomiasis in Rural and Semi-Urban Areas of the Moyen-Ogooué Province, Gabon

**DOI:** 10.3390/tropicalmed7010001

**Published:** 2021-12-22

**Authors:** Jean Claude Dejon Agobé, Henry Curtis Kariuki, Jeannot Fréjus Zinsou, Yabo Josiane Honkpehedji, Martin Peter Grobusch, Ayola Akim Adegnika

**Affiliations:** 1 Centre de Recherches Médicales de Lambaréné (CERMEL), Lambaréné P.O. Box 242, Gabon; jcagobe@cermel.org (J.C.D.A.); zinaff@gmail.com (J.F.Z.); hyjosy@gmail.com (Y.J.H.); m.p.grobusch@amsterdamumc.nl (M.P.G.); 2 Center of Tropical Medicine and Travel Medicine, Department of Infectious Diseases, Division of Internal Medicine, Amsterdam University Medical Centers, Location AMC, University of Amsterdam, P.O. Box 22660, 1100 DD Amsterdam, The Netherlands; 3 School of Medicine and Health Sciences, Kenya Methodist University (KeMU), P.O. Box 267, Meru 60200, Kenya; hckariuki@yahoo.com; 4 Leiden Medical University Centre, University of Leiden, Albinusdreef 2, 2333 ZA Leiden, The Netherlands; 5 Institut für Tropenmedizin, Eberhad Karls Universität Tübingen, Wilhelmstraße 27, 72074 Tübingen, Germany; 6 German Centre for Infection Research (DZIF), Partner Site Tübingen, Wilhelmstraße 27, 72074 Tübingen, Germany; 7 German Centre for Infection Research (DZIF), African Partner Institution, CERMEL, Lambaréné P.O. Box 242, Gabon

**Keywords:** schistosomiasis, *Schistosoma haematobium*, Gabon, *Bulinus* spp., cercarial shedding

## Abstract

The objective of this pilot malacological survey was to identify the snail intermediate hosts for *Schistosoma* *haematobium* in endemic rural and semi-urban areas of Gabon. Snails were collected, morphologically identified, and tested for infection by cercarial shedding. Released cercariae were morphologically identified using low-power light microscopy. A total of six species of snails were collected throughout the study area, with *Bulinus* *truncatus*, *B. forskalii*, and *Potadoma* spp. being the most predominant species collected. Only the *Bulinus* species were tested for infection by cercarial shedding, of which only *B. truncatus* shed cercariae. Some *B. truncatus* shed mammalian schistosome cercariae, while others shed *Gymnocephalus* cercariae. Our results indicate that *B. truncatus* appears to be a potential intermediate host of schistosomiasis in Gabon, where cases of *S. haematobium*, *S. guineensis,* and *S. intercalatum* infection are reported. However, it will be important to further understand the species diversity and transmission dynamics of schistosomes.

## 1. Introduction

Schistosomiasis, a water-borne helminthic disease, is the second most important parasitic infection after malaria in terms of public health and economic impact [1]. Human infections are caused by three main species of flukes, namely, *Schistosoma haematobium* causing urogenital schistosomiasis, and *S. japonicum* and *S. mansoni*, which both cause intestinal schistosomiasis. There are other species that cause intestinal schistosomiasis, although their distribution is restricted to specific foci, including *S. guineensis* and its variant *S. intercalatum* in Central Africa, and *S. mekongi* in South East Asia [2]. The worldwide geographical distribution of the different *Schistosoma* species depends on the presence and distribution of their freshwater snail intermediate hosts; the snail genus is specific to the species of the parasite, with some variations across countries. In Africa, for instance, predominantly snails of the genus *Biomphalaria* serve as intermediate hosts of *S. mansoni*, while snails of the genus *Bulinus* serve as intermediate hosts of *S. haematobium*, as well as of *S. intercalatum* and *S. guineensis* [3]. *Bulinus* spp. are also known as the intermediate hosts of *S. bovis* [4], a schistosome parasite of ruminants such as cattle, goats, sheep, and pigs. The geographical distribution and density of the snail population and their dynamics over time relate to the epidemiological situation of the disease in a particular human population, rendering schistosomiasis a focal disease. 

Freshwater snail control is part of the WHO’s recommendation for the control of schistosomiasis [2]. Malacological data is therefore essential for a better understanding of the disease transmission, but also for the implementation of a proper and adequate schistosomiasis control program. Gabon is a central African country located on the equator. Although the region is known to be endemic for schistosomiasis, very few malacological data are available for the country, and most of it is historic. More recent data by Mintsa et al. (2009) reported the presence of *B. globosus* and *B. forskalii* in two different sites in the Estuaire province; Libreville and Ekouk [5]. We conducted a pilot survey in rural and semi-urban areas located central to the country, known to be endemic for urogenital schistosomiasis, with the aim to provide basic information on the snails as intermediate hosts for schistosomiasis and on molluscan diversity in the Moyen-Ogooué, one of the nine provinces of Gabon.

## 2. Materials and Methods

**Figure 1:**
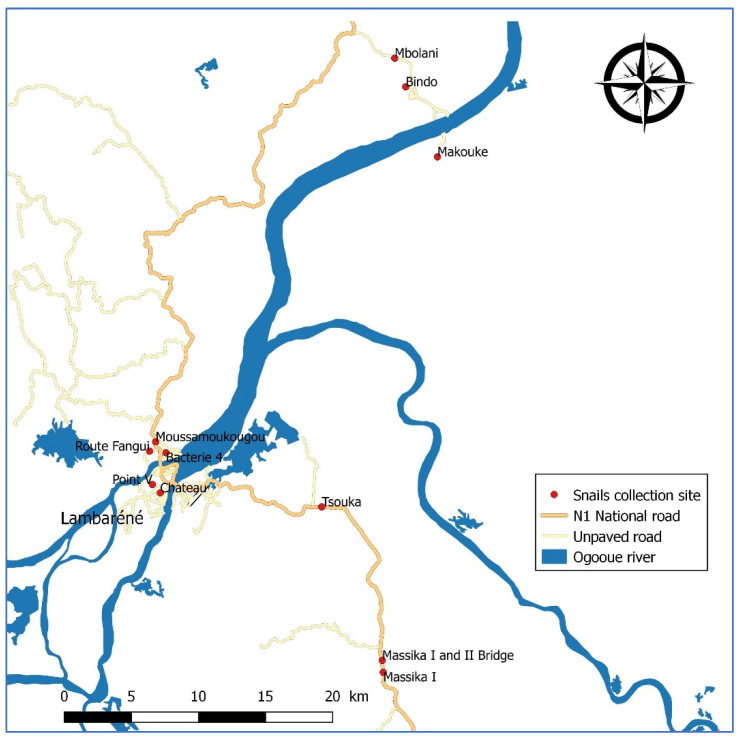
Distribution of the human-freshwater contact points selected for snail collection over the study area.

The surveys were carried out at CERMEL [6] and were conducted from 15–19 November 2013 on three different locations: Lambaréné, the provincial capital of the Moyen-Ogooué; the Zilé-PK area, which is a string of villages along the national road (RN1) south of Lambaréné from PK8 to PK33, including Tsouka and Massika I and II villages; and in Mbolani, namely the Bindo-Makouké villages, which is a remote area 65 km from Lambaréné by road (Figure 1). All these locations are either close to the Ogooué river, or are irrigated by its tributaries, with many lakes and swamps. In the region, the vegetation is made up of rainforests, and the weather is characterized by four seasons, long rainy (February to May) and dry (June to September) seasons, followed by short rainy (October to mid-December) and dry (mid-December and January) seasons. These areas are known to be schistosomiasis-endemic, with *S. haematobium* being the predominant species [7,8,9]. Indeed, we reported, in 2020, a 26% schistosomiasis prevalence in Lambaréné [9], while a prevalence of around 45% and 15% were reported earlier in 2014 and 2018 for the Nzilé-PK area and Bindo village, respectively [7,8].

For each of the three study areas, human-water contact sites, known as potential schistosomiasis foci, were identified. All sites had on average up to 50% vegetation cover, with the watercourse bed being either muddy, sandy, or both. At the selected sites, snails were collected systematically by three collectors for about ten minutes between 8 a.m. and 11 a.m. from aquatic plants and other objects in the habitats. Specifically, vegetation and any materials such as discarded pieces of clothing and tires were thoroughly searched for possibly attached mollusks. During the snail collections, the geographic coordinates of the site were taken using a hand-held GPS, and human-water contact behaviors were observed. All collected snails were placed in a perforated container with wet cotton wool or wet vegetation before being transported back to the CERMEL laboratory.

At the laboratory, snails were separated and identified mostly to the genus level based on the shell morphological characteristics using the standardized taxonomic keys proposed by the WHO identification center [10]. On the day of collection, snails were individually placed in a well plate for cercariae shedding, and dechlorinated clean commercial drinking water was added. The plate was covered to prevent snails from escaping but opened and closed regularly for air circulation. The plate was placed in indirect daylight and left for about three hours from noon to 3 p.m., and then it was examined. The wells were examined under a low-powered microscope for evidence of any emitted cercariae, which were then morphologically differentiated using standardized taxonomic keys [11].

## 3. Results

### 3.1. Snail Collection and Species Distribution

A number of snail collection points were selected over the study area (Figure 1). In the Zilé-PK area, the first area visited was Tsouka village, where six water contact sites that appeared as potential transmission hotspots were selected along a tributary of the Ogoouée River, namely, Mikoli River. Other sites that were visited included various sites in Massika I and Massika II villages. Within Lambaréné, snails were collected in small streams of some neighborhoods; Château, Fanguy, and Moussamoukougou, respectively. In Mbolani, Bindo, and Makouké villages, a total of four collection sites were targeted, as these were known as the main human-water contact points.

In total, six snail species were collected from a number of collection points. The overall freshwater snails that were found were: *Potadoma* species (most likely *P. freethi*), *Bulinus truncatus*, *Bulinus forskalii*, *Melanoides* species (most likely *M. tuberculata*), *Lanistes* (most likely *L. nsedweensis*), and *Gabiella* species. Table 1 presents the distribution of snail species collected in each study site. With regard to the *Bulinus* species, a total of 44 snails were collected over the study area, including four *B. forskalii* and 40 *B. truncatus*.

### 3.2. Cercarial Shedding

**Figure 2:**
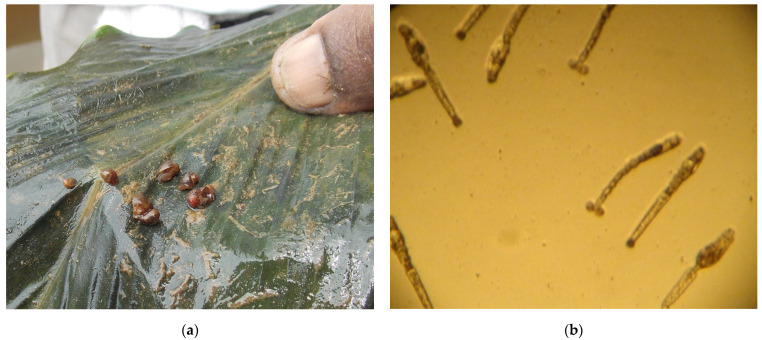
(**a**) Some *Bulinus truncatus* snails collected at Tsouka village; (**b**) Microscopic view of shedding of some mammalian (forked tail) and *Gymnocephalus* (single tail) cercariae.

When testing for cercarial shedding, none of the four *B. forskalii* snails examined were infected, while 12 (30%) of the 40 *B. truncatus* (Figure 2a) examined shed mammalian *Schistosoma* cercariae (Figure 2b), whilst others shed *Gymnocephalus* cercariae. *Bulinus* snails that shed schistosome cercariae were collected only in the Mikoli River of Tsouka village (Zilé-PK area).

## 4. Discussion

The present survey adds malacological information to the scarce data available from the schistosomiasis-endemic region. Our results establish the first evidence of cercarial shedding in the Moyen-Ogooué province. Indeed, we found that *B. truncatus* appears to be an intermediate host of schistosomiasis in the region. It is known that some *Bulinus* snails may act as intermediate hosts of *Schistosoma bovis* [4,12] which cannot be separated from *S. haematobium* by cercariae morphology. However, no domestic animals were observed at the study sites, nor any evidence of bovine game. Moreover, no data are available on the potential presence of *S. bovis* in Gabon, and particularly in the study area. Since the study area is known to be endemic for *S. haematobium* [7,8,9], we therefore strongly suspect that the mammalian cercariae were actually *S. haematobium* cercariae, shed by *B. truncatus*. However, the use of molecular tools to accurately identify *B. truncatus* as a snail host for *S. haematobium* cercariae in the area remains. As some cases of Schistosome eggs in stool have been reported in the region [9], and cases of *S. guineensis* have been reported in the country, the role of *B. truncatus* in the transmission of *S. intercalatum* and *S. guineensis* [13] in the country has to be further investigated.

The study was conducted in November, corresponding with the beginning of the rainy season. During the surveys, we observed a low density of snails in the study areas, particularly in the Bindo-Makouké villages. Since seasonal rainfall affects snail density [14], we hypothesize that this reflects the snail population density usually observed during the rainy season. Despite the low density of snails observed, the genus *Bulinus* was present in all three study areas, while *B. truncatus* was found in Lambaréné and in the Zilé-PK areas, known as areas with a moderate or high prevalence of urogenital schistosomiasis [7,8,9], compared to Bindo-Makouké, where the prevalence of the disease is low [7,8]. This suggests that the distribution of *B. truncatus* could sustain the prevalence of schistosomiasis in the region, and probably in the country. 

When exposed to daylight illumination, only *B. truncatus* shed cercariae. Similar to what was reported earlier by Mintsa et al. [5], no *B. forskalii* we collected shed cercariae. However, we found that a high proportion of *B. truncatus* shed cercariae (around 30%), particularly those from the Zilé-PK rural area. This is in contrast to what was reported from southern Mauritania and western Kenya where no to few (1.8%) snails sampled shed cercariae [15,16], respectively. Similarly, the number of cercariae shed by most of the snails was considerably higher than what is usually reported from other naturally infected snails. These results suggest that the *B. truncatus* intermediate hosts we identified are a very efficient vector of schistosomiasis in our study area, which contrasts with the observation of a similar snail species in Kenya, which is refractive to the local *S. haematobium* [17].

In addition to the *Bulinus* snails involved in schistosomiasis transmission, we found other snail intermediate hosts that are capable of transmitting other parasitic diseases. *Potadoma* spp. was one of the snail genera found, particularly in the Zilé-PK area. It has been suspected that *Potadoma* snails may be the intermediate hosts of the lung flukes of the human *Paragonimus* species (most likely *P. africanus* or *P*. *uterobilateralis*), which are reported to occur in parts of Central (Zaire and Cameroon) and West (Nigeria) Africa, respectively [18]. It would be of interest to clarify the role of this snail in Gabon, and particularly in Lambaréné and its surroundings, where some cases of paragonimiasis have already been reported [19,20].

## 5. Conclusions

*Bulinus* spp., a potential intermediate host of schistosomiasis, appears to be present in Gabon, particularly *B. globosus*, *B forskalii*, and, as we reported, *B. truncatus,* which appears to be an efficient intermediate host of schistosomiasis. However, it remains necessary to properly identify the species in Lambaréné and the surrounding areas using molecular analyses to understand the seasonality of snail transmission and population dynamics to guide an appropriate strategy for schistosomiasis control.

## Figures and Tables

**Table 1:** Distribution of snail species collected by study area.

Study Area	Snail Genus	Snail Species
Zilé-PK area; Tsouka, Massika I and Massika II villages	*Bulinus*	*B. truncatus*
	*Potadoma*	*P. freethi* ^1^
	*Melanoides*	*M. tuberculate* ^1^
	*Lanistes*	*L. nsedweensis* ^1^
Lambaréné town	*Bulinus*	*B. truncatus*
		*B. forskalii*
Mbolani, Bindo, and Makouké villages	*Bulinus*	*B. forskalii*
	*Gabiella*	*Gabiella* spp.

^1^ Most likely.

## Data Availability

Not applicable.

## References

[1] WHO Water-Related Diseases. http://www.who.int/water_sanitation_health/diseases-risks/diseases/schisto/en/.

[2] World Health Organization Fact Sheet: Schistosomiasis. http://www.who.int/news-room/fact-sheets/detail/schistosomiasis.

[3] World Health Organization Freshwater Snails. Vector.

[4] Adriko M., Tinkitina B., Tukahebw E.M., Standley C.J., Stothard J.R., Kabatereine N.B. (2018). The epidemiology of schistosomiasis in Lango region Uganda 60 years after Schwetz 1951: Can schistosomiasis be eliminated through mass drug administration without other supportive control measures?. Acta Trop..

[5] Nguema R.M., Milama K.M.N., Kombila M., Richard-Lenoble D., Tisseyre P., Ibikounlé M., Moné H., Mouahid G. (2010). Morphometric and molecular characterizations of schistosome populations in Estuaire province Gabon. J. Helminthol..

[6] Ramharter M., Agnandji S.T., Adegnika A.A., Lell B., Mombo-Ngoma G., Grobusch M.P., Matthew M., Muranaka R., Kreidenweiss A., Velavan T.P. (2021). Development of sustainable research excellence with a global perspective on infectious diseases: Centre de Recherches Médicales de Lambaréné (CERMEL), Gabon. Wien Klin Wochenschr..

[7] Ateba Ngoa U., Zinsou J.F., Kassa R.F.K., Ngoune Feugap E., Honkpehedji Y.J., Massinga-Loembe M., Kenguele M.H., Nkoma Mouima A.M., Mbenkep L.H., Wammes L.J. (2014). Assessment of the effect of *Schistosoma haematobium* co infection on malaria parasites and immune responses in rural populations in Gabon: Study protocol. SpringerPlus.

[8] Dejon-Agobé J.C., Zinsou J.F., Honkpehedji Y.J., Ateba-Ngoa U., Edoa J.-R., Adegbite B.R., Mombo-Ngoma G., Agnandji S.T., Ramharter M., Kremsner P.G. (2018). *Schistosoma haematobium* effects on *Plasmodium falciparum* infection modified by soil-transmitted helminths in school-age children living in rural areas of Gabon. PLoS Negl. Trop. Dis..

[9] Dejon-Agobé J.C., Honkpehedji Y.J., Zinsou J.F., Edoa J.-R., Adégbitè B.R., Mangoula A., Agnandji S.T., Mombo-Ngoma G., Ramharter M., Kremsner P.G. (2020). Epidemiology of schistosomiasis and soil-transmitted helminth co-infections among school children living in Lambaréné, Gabon. Am. J. Trop. Med. Hyg..

[10] WHO Snail Identification Centre—Danish Bilharziasis Laboratory (1973). A Field Guide to African Freshwater Snails. 3: North East African Species.

[11] Frandsen F., Christensen N.O. (1984). An introductory guide to the identification of cercariae from African freshwater snails with special reference to cercariae of trematode species of medical and veterinary importance. Acta Trop..

[12] Southgate V.R., Brown D.S., Rollinson D., Ross G.C., Knowles R.J. (1985). *Bulinus tropicus* from Central Kenya acting as a host for *Schistosoma bovis*. Z. Parasitenkd.

[13] Tian-Bi Y.-N.T., Webster B., Konan C.K., Allan F., Diakité N.R., Ouattara M., Salia D., Koné A., Kakou A.K., Rabone M. (2019). Molecular characterization and distribution of Schistosoma cercariae collected from naturally infected bulinid snails in northern and central Côte d’Ivoire. Parasit. Vectors.

[14] Odongo-Aginya E.I., Kironde F.K., Kabatereine N.B., Kategere P., Kazibwe F. (2008). Effect of seasonal rainfall and other environmental changes, on snail density and infection rates with *Schistosoma mansoni* fifteen years after the last snails’ study in Kigungu, Entebbe, Uganda. East Afr. Med. J..

[15] Opisa S., Odiere M.R., Jura W.G., Karanja D.M., Mwinzi P.N. (2011). Malacological survey and geographical distribution of vector snails for schistosomiasis within informal settlements of Kisumu City, western Kenya. Parasit. Vectors.

[16] Gbalégba N.G.C., Silué K.D., Ba O., Ba H., Tian-Bi N.T.Y., Yapi G.Y., Kaba A., Koné B., Utzinger J., Koudou B.G. (2017). Prevalence and seasonal transmission of *Schistosoma haematobium* infection among school-aged children in Kaedi town, southern Mauritania. Parasit. Vectors.

[17] Brown D.S., Shaw K.M. (1989). Freshwater snails of the *Bulinus truncatus/tropicus* complex in Kenya: Tetraploid species. J. Molluscan. Stud..

[18] WHO WHO|Paragonimiasis. http://www.who.int/foodborne_trematode_infections/paragonimiasis/en/.

[19] Malvy D., Ezzedine K.H., Receveur M.C., Pistone T., Mercié P., Longy-Boursier M. (2006). Extra-pulmonary paragonimiasis with unusual arthritis and cutaneous features among a tourist returning from Gabon. Travel Med. Infect. Dis..

[20] Sachs R., Kern P., Voelker J. (1983). Paragonimus uterobilateralis as the cause of 3 cases of human paragonimiasis in Gabon. Tropenmed. Parasitol..

